# 873 Development of Decubitus Ulcers Following Burn Injury: A National Database Study

**DOI:** 10.1093/jbcr/iraf019.404

**Published:** 2025-04-01

**Authors:** Hilary Liu, Rebecca Hohsfield, José Arellano, Mare Kaulakis, Christopher Fedor, Daniel Najafali, Garth Elias, Alain Corcos, Jenny Ziembicki, Francesco Egro

**Affiliations:** University of Pittsburgh Medical Center; University of Pittsburgh Medical Center; University of Pittsburgh Medical Center; University of Pittsburgh School of Medicine; University of Pittsburgh School of Medicine; Carle Illinois College of Medicine; University of Pittsburgh Medical Center; University of Pittsburgh Medical Center; University of Pittsburgh Medical Center; University of Pittsburgh Medical Center

## Abstract

**Introduction:**

Burn patients are at increased risk of developing decubitus ulcers due to prolonged immobilization and systemic complications during extended hospital stays. These ulcers can lead to significant complications, such as delayed wound healing, increased morbidity, and prolonged hospitalizations. This national database study characterizes burn patients who developed decubitus ulcers.

**Methods:**

A retrospective review was performed using data from the ABA National Registry from January 2013 to December 2016 on burn patients who developed decubitus ulcers. Patient demographics, burn characteristics, clinical course, and hospitalization data were extracted and analyzed using descriptive statistics.

**Results:**

Of 106,956 patients in the database, 257 developed decubitus ulcers during their hospital stay. Patients were 73.2% male and 26.8% female, with a mean age of 51.8 ± 21.2 years. Diabetes mellitus was present in 41 (16.0%) patients. The most common burn etiology was flame (n=122; 47.5%), followed by scald (n=33; 12.8%). The mean total body surface area (TBSA) affected was 25.2%±21.2%. Patients had a mean hospital stay of 49.7±39.3 days, with 32.2±32.3 days in the intensive care unit and 22.2±24.9 days on ventilator support. Regarding discharge disposition, 147 patients (57.2%) were transferred to other facilities, 66 (25.7%) were discharged home under various circumstances, and 35 (13.6%) expired during their hospital stay.

**Conclusions:**

This study highlights key aspects of burn patients who develop decubitus ulcers, a rare but serious complication. Prolonged hospital stays, ICU admissions, and ventilator use reflect the complexity of these cases. The high mortality rate and frequent transfers for ongoing care further underscore the severity. The findings stress the importance of prevention, early detection, and aggressive treatment.

**Applicability of Research to Practice:**

This study provides valuable data for burn care research, highlighting the need for targeted prevention and management strategies for decubitus ulcers in burn patients. The findings offer a basis for future research to explore specific risk factors and improve care protocols, ultimately reducing the incidence and severity of these complications.

**Funding for the Study:**

N/A

